# Synthesis and Characterization of Core–Double-Shell-Structured PVDF-*grafted*-BaTiO_3_/P(VDF-*co*-HFP) Nanocomposite Films

**DOI:** 10.3390/polym15143126

**Published:** 2023-07-22

**Authors:** Fatima Ezzahra Bouharras, Salima Atlas, Simone Capaccioli, Massimiliano Labardi, Abdelghani Hajlane, Bruno Ameduri, Mustapha Raihane

**Affiliations:** 1 IMED-Lab., Faculty of Sciences and Techniques, Cadi Ayyad University (UCA), Av. A. El Khattabi, B.P. 549, Marrakesh 40000, Morocco; fz.bouharras@gmail.com (F.E.B.); salimat2703@gmail.com (S.A.); ahajlane@gmail.com (A.H.); 2 Dipartimento di Fisica, Università di Pisa, Largo Pontecorvo 3, 56127 Pisa, Italy; simone.capaccioli@unipi.it; 3 ICGM, Université de Montpellier, CNRS, ENSCM, 34293 Montpellier, France; bruno.ameduri@enscm.fr; 4 Polydisciplinary Faculty, Sultan Moulay Sliman University, Mghila, P.O. Box 592, Béni-Mellal 23000, Morocco; 5 CNR-IPCF, Sede Secondaria di Pisa, c/o Dipartimento di Fisica, Università di Pisa, Largo Pontecorvo 3, 56127 Pisa, Italy; 6 CISUP, Centro per l’Integrazione della Strumentazione dell’Università di Pisa, Lungarno Pacinotti 43/44, 56126 Pisa, Italy

**Keywords:** dielectric properties, interfacial polarization, nanoparticles, fluoropolymers, polymer composites

## Abstract

Core–double-shell-structured nanocomposite films consisting of polyvinylidene fluoride-*grafted*-barium titanate (PVDF-*g*-BT) incorporated into a P(VDF-co-hexafluoropropylene (HFP)) copolymer matrix were produced via a solution mixing method for energy storage applications. The resulting films were thoroughly investigated via spectroscopic, thermal, and morphological analyses. Thermogravimetric data provided an enhancement of the thermal stability, while differential scanning calorimetry indicated an increase in the crystallinity of the films after the addition of PVDF-*g*-BT. Moreover, broadband dielectric spectroscopy revealed three dielectric processes, namely, glass–rubber relaxation (α_a_), relaxation associated with the polymer crystalline phase (α_c_), and slower relaxation in the nanocomposites resulting from the accumulation of charge on the interface between the PVDF-*g*-BT filler and the P(VDF-*co*-HFP) matrix. The dependence of the dielectric constant from the composition was analyzed, and we found that the highest permittivity enhancement was obtained by the highest concentration filler added to the largest concentration of P(VDF-*co*-HFP). Mechanical analysis revealed an improvement in Young’s modulus for all nanocomposites versus pristine P(VDF-*co*-HFP), confirming the uniformity of the distribution of the PVDF-*g*-BT nanocomposite with a strong interaction with the copolymer matrix, as also evidenced via scanning electron microscopy. The suggested system is promising for use in high-energy-density storage devices as supercapacitors.

## 1. Introduction

Dielectric nanocomposites have gained significant interest for their extensive applications in energy storage systems. These composites combine the high permittivity of perovskite oxides like BaTiO_3_ with the desired characteristics of polymers, such as fracture toughness, flexibility, and ease of processing [1,2,3,4]. Specifically, ferroelectric fluorinated polymers have attracted considerable attention for high-tech applications. Examples include polyvinylidene fluoride (PVDF) and its copolymers, like poly(vinylidene fluoride-*co*-trifluoroethylene) (P(VDF-*co*-TrFE)), poly(vinylidene fluoride-*co*-chlorotrifluoroethylene) (P(VDF-*co*-CTFE)), poly(vinylidene fluoride-*co*-hexafluoropropylene) (P(VDF-*co*-HFP)), and the terpolymer P(VDF-*ter*-TrFE-*ter*-CTFE). These polymers have drawn attention because, among all polymeric materials, they have the greatest dielectric constants (ε′∼10–12 at 1 kHz) [5,6,7,8,9,10,11], which are a result of their strong C-F dipole moment [12].

In a comprehensive study conducted in 2016 [13], the potential usage of ferroelectric VDF copolymers and BaTiO_3_ nanoparticles in dielectric nanocomposite materials for energy storage applications, such as high-energy-density capacitors, was examined. To achieve satisfactory dielectric constant nanocomposites, high filler loading of ferroelectric BaTiO_3_ nanoparticles (e.g., >50 vol %) is necessary due to their significantly higher permittivity compared to the matrix material. However, the aggregation and phase separation of BaTiO_3_ within the polymer matrix are caused by the significant discrepancy in surface energy and electrical characteristics between the nanofillers and PVDF matrix materials. The complicated interfacial polarization and the unequal distribution of the electric field close to the nanofiller–matrix interfaces have a negative impact on the energy density, causing it to decrease [11,14,15].

Making sure that the nanoparticles are spread out evenly in the polymer matrix has been a long-term goal in the development of excellent dielectric storage materials. The process can be performed by modifying the surface and functionalizing the nanoparticles, thereby improving their compatibility with the polymer matrix. Various methods have been explored for surface modification, including hydroxylation [16], phosphonic acid treatment [17,18,19], dopamine coating [11,20], decoration with Ag nanocrystals [21], and the use of coupling agents [22,23,24]. In a different method known as “interfacial modifier engineering”, polymers are grafted onto the surface of BaTiO_3_ nanoparticles to create core–shell nanostructures [25,26,27,28]. This procedure seeks to chemically alter the surface of BaTiO_3_ using surface-initiated in situ regulated radical polymerization (RDRP) techniques such atom transfer radical polymerization (ATRP) and reversible addition–fragmentation chain transfer (RAFT). The modified nanoparticles are subsequently mixed into PVDF-based polymers to create BaTiO_3_-*g*-polymers@PVDF copolymers, that is, a core–double-shell system [25,26,27,29,30]. For instance, Du et al.’s [27] ATRP-functionalized core–shell, BaTiO_3_-grafted P(tBuA) nanoparticles were added to a PVDF matrix after being functionalized with poly(tert-butyl acrylate) P(tBuA) on the BaTiO_3_ surface. The nanocomposites that were obtained had better dielectric permittivity than BaTiO_3_-based PVDF films that had not been functionalized. With effective dielectric constants of 26.5 and 20.4 at 150 MV/m for PVDF films with a 30 wt % loading of BaTiO_3_-*g*-P(tBuA) and pure PVDF films, respectively, the improvement in dielectric permittivity was relatively marginal. Weak interactions between PVDF polymers and non-fluorinated modified nanoparticles resulted in structural defects in nanocomposite films containing more than 30% nanoparticles by weight. These structural flaws lead to low mechanical strength.

To improve the interactions between nanofillers and the fluoropolymer matrix at the interface, fluorinated polymers have been added to the surface of BaTiO_3_ nanoparticles using a solution blending method. Various fluorinated polymers, including poly(2,2,2-trifluoroethyl methacrylate) [27], poly(1H,1H,2H,2H-perfluorooctyl methacrylate) [31], poly(2,2,2-trifluoroethyl acrylate), poly(1H,1H,2H,2H-heptadecafluorodecyl acrylate) [27], poly(2,5-bis[(4-trifluoromethoxyphenyl) oxycarbonyl] styrene) [4,32], and poly(dodecafluoroheptyl methacrylate) [33], have been utilized for this purpose. Strong interchain forces are produced when polymers based on PVDF are closely attached onto the fluorinated surfaces of nanoparticles. Zhang et al. [26], for instance, investigated the effects of two-fluorinated shell interactions on the characteristics of nanocomposites. Poly(methyl methacrylate) (PMMA) or poly(2,2,2-trifluoroethyl methacrylate) (PTFEMA) were grafted onto BaTiO_3_ in the first step utilizing ATRP polymerization. The modified nanoparticles were then mixed with a solution and added to the PVDF matrix. The enhancement in dielectric permittivity was seen in both systems. Notably, compared to the system using PMMA-modified nanoparticles, the nanocomposites produced when the fluorinated polymer (PTFEMA) was utilized for nanoparticle functionalization displayed high dielectric constants and minimal dielectric losses. This improvement was ascribed to the robust forces that interacted with the matrix and the two-fluorinated shell. A double-shell nanocomposite was created [34] using two distinct polymers, in opposition to the previously reported fluorinated poly(meth)acrylates grafted onto a BaTiO_3_ shell. As a grafting-on approach, the surface of BaTiO_3_ was first functionalized with either polystyrene or PVDF using a thiol-ene process. After this, a second PVDF shell was added using hot pressing and casting. The outcomes demonstrated that functionalizing BaTiO_3_ with PVDF led to a significant improvement in the dielectric constant and a reduction in dielectric loss compared to the nanocomposites prepared using core–shell PS-*g*-BaTiO_3_. In fact, PVDF-*g*-BT/PVDF exhibited a 15% increase in its dielectric constant versus PS-*g*-BT/PVDF at 30 wt% load charging, owing to the similarity in structure between the filler shell and the host polymer matrix. In addition, to increase the compatibility of VDF with fluoropolymers, our research team, in a recent study, utilized in situ RAFT polymerization of VDF from modified BaTiO_3_ nanoparticles [35].

Lately, core–double-shell structures consisting of polymers and PVDF-based co-polymers and ter-polymers grafted onto BaTiO_3_ nanoparticles have been reported [36]. Although PVDF is the most widely studied double-shell fluoropolymer, it has certain disadvantages, including a high melting temperature that increases the energy costs of processing and low solubility in usual organic solvents [12,36]. The copolymerization of VDF is a widely used and successful method to systematically change polymer characteristics to bypass these restrictions. It is commonly used to create fluorinated copolymers that are sold commercially. We chose to employ the ferroelectric copolymer P(VDF-*co*-HFP) in this work. Due to PVDF’s crystallinity and exceptional mechanical stability, P(VDF-*co*-HFP) is widely used in a variety of industries, including in coatings, solar fabrics, and especially in lithium-ion batteries as a cathode binder or membrane polymer [5,6,7,8,9,10,37,38,39]. However, its promise as an insulating material is constrained by its low permittivity (6–8) in comparison to PVDF and several other copolymers and terpolymers. Limited studies have focused on developing core–double-shell structures of fluorinated polymer-g-BT using P(VDF-*co*-HFP) [27].

This study focuses on a core–double-shell system comprising P(VDF-*co*-HFP) as the polymer matrix and PVDF-*g*-BaTiO_3_ as the filler. The previously described RAFT polymerization of VDF was used to create the core–shell-structured PVDF-*g*-BaTiO_3_ nanocomposites [35]. Subsequently, these nanocomposites were incorporated into a copolymer matrix of P(VDF-*co*-HFP) using the solution blending method. This approach offers several advantages. The P(VDF-*co*-HFP) matrix and the insulating fluoropolymer shells (PVDF-*g*-BT) have identical chemical structures and surface energies. So, not only does it become easier for BaTiO_3_ nanoparticles to spread out, but it also makes it easier for the nanoparticles to stick to the fluoropolymer matrix compared to modified BaTiO*3* nanoparticles that have not been fluorinated. Consequently, the dielectric permittivity of the P(VDF-*co*-HFP) matrix is enhanced [26]. Furthermore, Size-Exclusion Chromatography analysis of grafted PVDF in modified BT revealed lower molar masses (6500 g mol^−1^ < *M*_n_ < 7500 g mol^−1^) [35], which led to poor film-forming properties and brittle materials. However, when casting films using commercial P(VDF-*co*-HFP) copolymer in core–double-shell nanocomposites, mechanically stable films were obtained. These properties are crucial for developing nanocomposite films using molding techniques for energy storage devices. In this study, the PVDF-*g*-BaTiO_3_/P(VDF-*co*-HFP) films were extensively characterized using FT-IR, SEM, TGA, and DSC. In addition, the dielectric and mechanical properties were explored, and the impact of the PVDF-*g*-BaTiO_3_ nanocomposite fraction on the film characteristics was investigated.

## 2. Materials and Methods

### 2.1. Materials

Commercially available P(VDF-*co*-HFP) (Kynar Flex-3120, 3 mol% of HFP) copolymer in pellets was purchased from Arkema, France. The ^19^F NMR spectrum of this polymer is reported in Figure S1 of the Supporting Information. DMF solvent was provided by Fisher. The PVDF-*g*-BT nanocomposites used in the present study were synthesized via the reversible addition–fragmentation chain transfer (RAFT) polymerization of VDF in the presence of BT nanoparticles functionalized with a xanthate group (Scheme S1 in the Supporting Information). PVDF-*g*-BT nanocomposites with two different concentrations of BT were used for the preparation of our core–double-shell-structured samples. Such nanocomposites were produced through different loading (feed) of BT nanoparticles during production, namely 10% and 20% in weight; these fractions are used to label these nanocomposites in this work. However, the actual weight concentrations of BT nanoparticles grafted using PVDF chains were 16% and 38%, respectively, after the removal of ungrafted and physisorbed chains by washing them with acetone, as determined in a previous work by our team [40]. The HR MAS NMR ^19^F spectra of the two PVDF-*g*-BT nanocomposites employed are reported in Figures S2 and S3 of the Supporting Information [35]. TEM pictures of the BT particles grafted with PVDF chains were reported in Ref. [35], giving evidence of the core–shell structure.

### 2.2. Film Preparation

**Scheme 1:**
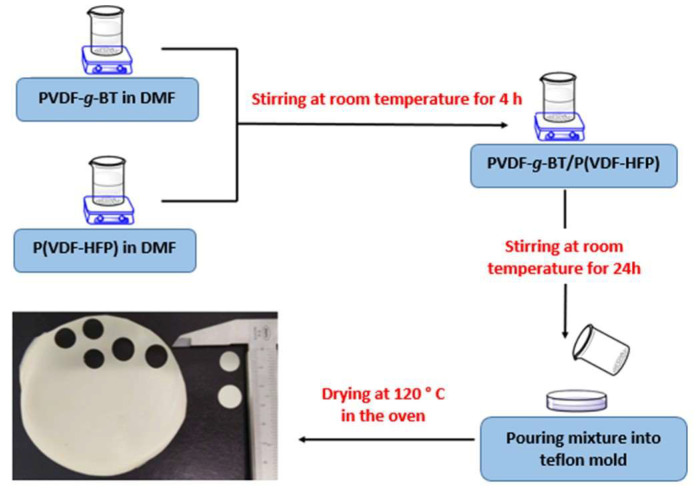
Preparation method of the nanocomposite films, and picture of the produced films.

The nanocomposite films were fabricated using the solution blending method with varying weight fractions of the components. The nomenclature employed in this study is presented in Table 1. For example, to prepare 10-10 nanocomposite films, 10 wt% (0.4 g) of the PVDF-g-BT nanocomposite, obtained from a 10% feed concentration, was dispersed in DMF (dimethylformamide) under ultrasonication for 4 h. Simultaneously, 90% by weight (3.6 g) of the P(VDF-*co*-HFP) copolymer was solubilized in DMF at room temperature for 4 h. Subsequently, the nanocomposite was slowly added to the copolymer solution while vigorously stirring the mixture at room temperature. The solution was prepared in an Erlenmeyer flask covered with a rubber septum. The resulting mixture was stirred for 24 h before being put into a Teflon mold and dried at 120 °C in an oven (Scheme 1).

### 2.3. Characterization

#### 2.3.1. Fourier Transform Infrared (FT-IR) Spectroscopy

A Perkin Elmer Spectrum 1000 FT-IR spectrometer with a universal Attenuated Total Reflection (ATR) accessory was used to collect the infrared spectra. A total of 16 scans were averaged at a resolution of 2 cm^−1^ while scanning the samples in transmittance mode between 4000 and 400 cm^−1^.

#### 2.3.2. Thermogravimetric Analysis (TGA)

Thermogravimetric measurements were conducted using TA Instruments Q50 apparatus. The samples were subjected to heating under an air atmosphere, which consisted of a mixture of nitrogen and oxygen, at flow rates of 60 mL/min and 40 mL/min, respectively. The heating rate employed was 20 °C/min, starting from room temperature and reaching a maximum temperature of 700 °C.

#### 2.3.3. Differential Scanning Calorimetry (DSC)

The films were subjected to the following heating and cooling cycles for DSC measurements using a Netzsch DSC 200 F3: initial heating from 20 °C to 200 °C at 20 °C/min; cooling from 200 to −70 °C at 20 °C/min; isothermal plateau at −70 °C for 10 min; second heating from −70 °C to 200 °C at 10 °C/min; and final cooling from 200 °C to 20 °C at 40 °C/min. The second heating/cooling cycles were used for the DSC thermograms that are provided in this study. Prior to analysis, the instrument’s calibration with noble metals was verified using an indium sample (T_m_ = 156.6 °C).

The area of the enthalpy peak and the point at the bottom of the valley with the greatest depth were used to calculate the melting temperatures and enthalpies (*H*_m_), respectively. The crystallinity level of the polymers, *χ*, was determined using the formula below:

(1) $$\chi [\%]=\frac{{\Delta H}_{m}}{{\Delta H}_{c}}\times 100$$

where Δ*H*_m_ is the melting enthalpy of the specimen under discussion, and Δ*H*_c_ (104.7 J g^−1^) refers to the melting enthalpy of a 100% crystalline pure α-phase PVDF, which is taken to be identical to that of P(VDF-*co*-HFP) [5,7].

#### 2.3.4. Scanning Electron Microscopy (SEM)

The microstructure of the PVDF-*g*-BT@P(VDF-*co*-HFP) nanocomposite films was examined using a scanning electron microscope (SEM) equipped with energy-dispersive mapping (EDS) capabilities (Zeiss HD15). To enhance conductivity, all samples were deposited onto a conductive adhesive carbon tape and subsequently coated with platinum. This preparation allowed for improved imaging and analysis of the samples.

#### 2.3.5. Dielectric Measurements

A Novocontrol Technologies GmbH & Co. (Montabaur, Germany) Alpha Analyzer spectrometer outfitted with a Novocontrol Quatro nitrogen gas flow cryostat was used to perform broadband dielectric spectroscopy (BDS). The dielectric measurements were conducted on round film portions (such as the ones shown in Scheme 1), which were placed between two parallel electrodes in the active cell. The sample thickness was in the range of 0.16 to 0.26 mm. Measurements were performed under a sinusoidal applied voltage of 1.5 V. Collecting the dielectric response while increasing the frequency *f* of the imposed sinusoidal voltage in the range of ~5·10^−2^ Hz to 10^7^ Hz, with logarithmic increments, at constant temperature, maintained for around 30 min, allowed us to obtain the isothermal spectra. The temperature range that was investigated was from −100 °C to 125 °C, with steps of 5 or 10 °C.

#### 2.3.6. Mechanical Analysis

Using an INSTRON 5566 tensile machine with a 2-kN load cell, the samples underwent uniaxial tensile testing at a crosshead speed of 2.5 mm/min. The stiffness of the films was determined by analyzing the stress-versus-strain curves within the linear region. Four rectangular specimens were used for each sample, with the film thickness ranging from 0.16 to 0.26 mm. Trendlines were employed to calculate the stiffness within a strain range of 0–2%.

## 3. Results and Discussion

Using FT-IR, SEM, TGA, and DSC, the produced PVDF-*g*-BaTiO_3_/P(VDF-*co*-HFP) films were thoroughly analyzed. The dielectric and mechanical properties were also investigated via BDS and tensile stress measurements, with a particular focus on the former ones, which are the most relevant for energy storage applications.

### 3.1. FT-IR Spectral Analysis

**Figure 1:**
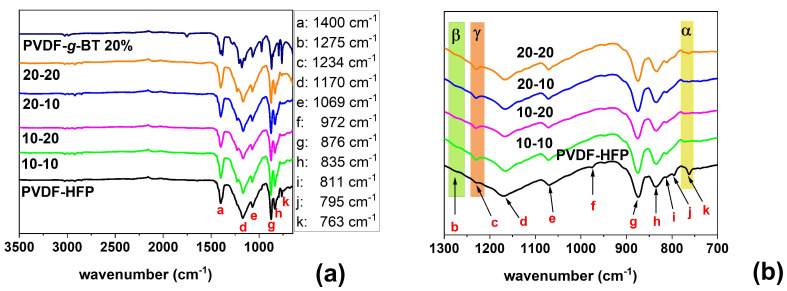
(**a**) FT-IR spectra of the P(VDF-*co*-HFP) copolymer and 10-10, 10-20, 20-10, and 20-20 nanocomposite films produced via the solution mixing method. The spectrum of the PVDF-*g*-BT core nanocomposite is also shown for comparison. (**b**) FT-IR spectral expansion of the 700–1300 cm^−1^ region.

The FT-IR spectra of the purest P(VDF-*co*-HFP) copolymer and the nanocomposite films 10-10, 10-20, 20-10, and 20-20 are shown in Figure 1. The band seen in the spectra at about 1062 cm^−1^ relates to the CF_2_’s symmetrical stretching mode. Additionally, the peaks found at 1170 cm^−1^ and 1400 cm^−1^ are the -C-F- groups inside the vinyl moiety’s scissoring and bending vibrations, respectively [6,7,9,41]. The assignment of FTIR bands to PVDF crystalline phases is often a subject of debate. In this study, we followed reference [41], which identifies specific bands for the α-phase (763 cm^−1^) as well as the two electroactive phases, β (1275 cm^−1^) and γ (1234 cm^−1^). Notably, the band at 763 cm^−1^ is observed in the copolymer but disappears in all nanocomposites. The same applies to the bands at 795 cm^−1^ and 972 cm^−1^, which are also characteristic of the crystalline α-phase. Both β-phase and γ-phase bands were already present in the copolymer (Figure 1b, bands “b” and “c”) but with minimal intensity. In the nanocomposites, the increase in the γ-phase is more pronounced compared to that in the β-phase. However, due to the limited crystallinity of the nanocomposites, these bands remain weak. Also, the amorphous phase of P(VDF-*co*-HFP) is clear from the band at 876 cm^−1^ (band “g” in Figure 1).

Upon the introduction of PVDF-*g*-BT nanocomposites into the P(VDF-*co*-HFP) matrix, the peaks associated with the α-phase structure (762, 794, and 972 cm^−1^) are eliminated. This shows that the inclusion of fillers in the P(VDF-*co*-HFP) matrix induces a transformation of the α-phase to another crystalline phase or to an amorphous state. These findings align with a study by Kumar et al. [42], where nanocomposite films were prepared using graphene oxide embedded in a P(VDF-*co*-HFP) matrix through a solution casting method. In their study, the FT-IR spectra also demonstrated the disappearance of characteristic peaks related to the α-phase structure in the nanocomposite. Moreover, the P(VDF-*co*-HFP) bands at 1400, 1069, and 876 cm^−1^ experienced slight shifts to 1402, 1071, and 875 cm^−1^, respectively, in the composite samples. These shifts observed in PVDF-*g*-BT/P(VDF-*co*-HFP) composite materials indicate the occurrence of PVDF-P(VDF-*co*-HFP) polymer interactions within the composite system.

### 3.2. Scanning Electron Microscopy (SEM)

**Figure 2:**
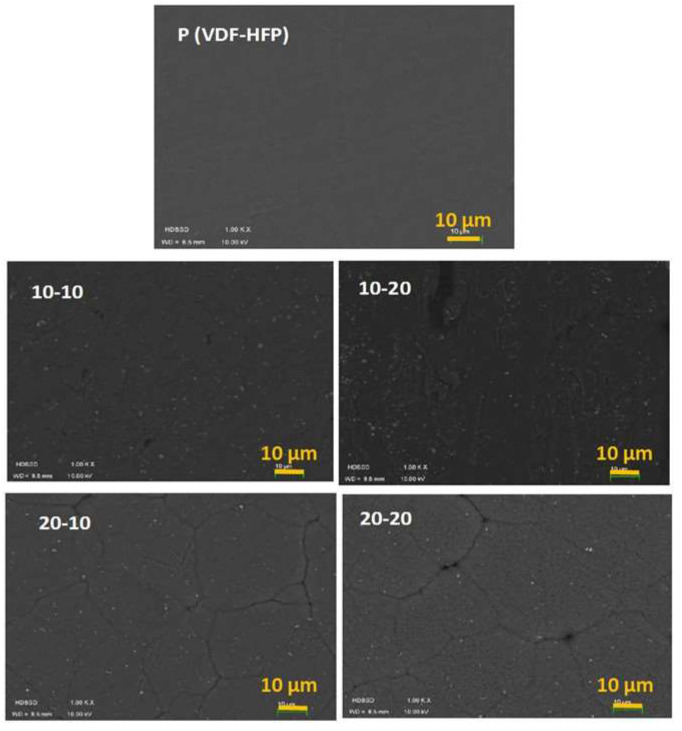
SEM micrographs of pure P(VDF-*co*-HFP) and its nanocomposite films with PVDF-*g*-BT.

Energy-dispersive spectroscopy (EDS) mapping and scanning electron microscopy (SEM) techniques were used for investigating the film’s morphology. The drying conditions used during preparation have a significant impact on the shape of the cast films [6,43]. In a work by Tian et al. [43], P(VDF-*co*-HFP) membrane material was created by dissolving P(VDF-*co*-HFP) pellets in a solvent under two different circumstances. The membranes that were dried under atmospheric conditions exhibited a porous structure, whereas those dried at 60 °C under vacuum showed a denser structure. In our investigation, the drying procedure was carried out under vacuum at 120 °C, producing dense sheets. The cross-section morphologies of the PVDF-*g*-BT/P(VDF-*co*-HFP) nanocomposite films and the pure P(VDF-*co*-HFP) copolymer are shown in Figure 2.

There are many nanoparticle fillers in the polymer. The PVDF-*g*-BT nanocomposites are clearly well integrated into the P(VDF-*co*-HFP) copolymer in all samples. In addition, no obvious aggregation, cavities, or flaws are seen in the films. These results suggest that the grafted PVDF polymer shell significantly improves the adhesion between the BT nanoparticles and the P(VDF-*co*-HFP) matrix, resulting in better PVDF-*g*-BT nanoparticle dispersion. Qian et al. [32], Ma et al. [34], and Jiang et al. [37], who studied the morphology of fluorinated BT-*g*-styrene/P(VDF-TrFE-CTFE) nanocomposites, double-shell PVDF-*g*-BT/PVDF composites, and P(VDF-*co*-HFP) composites with core-structured Fe_2_O_3_@BT nanofillers, provided the same findings.

Before SEM analysis, the PVDF-*g*-BT/P(VDF-*co*-HFP) core–double-shell nanocomposite films obtained after the casting were uniform, and no visible cracks were observed. One explanation could be that the electron-beam induces cracking in the composites’ thin films due to the evaporation of remaining solvent, as observed by Schneider et al. [44]. In this study, an original suspension had a small amount of immiscible liquid added to it. This additional liquid keeps the particles’ cohesiveness, changes the structure, speeds up drying, and reduces cracks. SEM was used to confirm the morphology before and after adding the immiscible liquid.

In addition, Yadavalli et al. [45] observed a curious cracking phenomenon in organic-inorganic halide perovskite thin films during SEM. This team reported that this phenomenon can be seen in numerous published SEM micrographs. These films served as a demonstration of the mechanisms causing this *e*-beam-induced damage, obviating the need for thorough SEM characterization and comprehension. Tensile stresses accumulate because of the *e*-beam-induced fast volatilization of the organic species from the surface of these films in the SEM, and these stresses are controlled by the thin-film grain size to cause grain boundary cracking.

**Figure 3:**
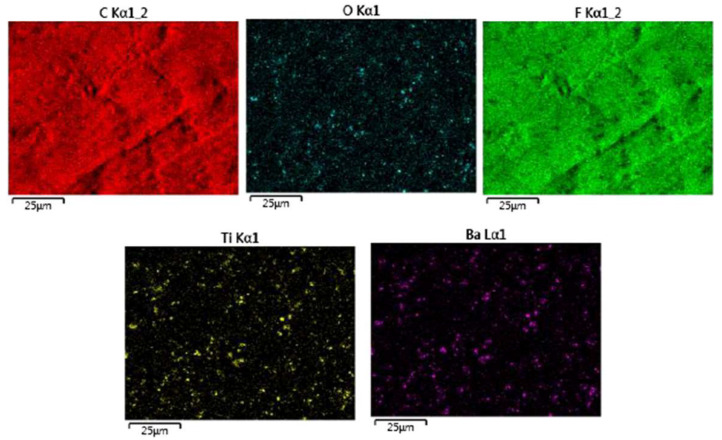
EDS elemental maps of the 10-10 nanocomposite for the C, O, F, Ti, and Ba elements.

To look at the spatial distribution of the nanoparticle elements, the EDS method was used. The elemental mapping of the 10-10 film (PVDF-*g*-BT 10% @ P(VDF-*co*-HFP) (10/90)) is shown in Figure 3. According to the findings, the sample contains elements of Ba, Ti, C, O, and F, which are also present in core–double-shell nanocomposite films. Additionally, the existence of Ba, Ti, and O components suggests that BT-modified nanoparticles have dispersed.

### 3.3. Thermogravimetric Analysis (TGA)

**Figure 4:**
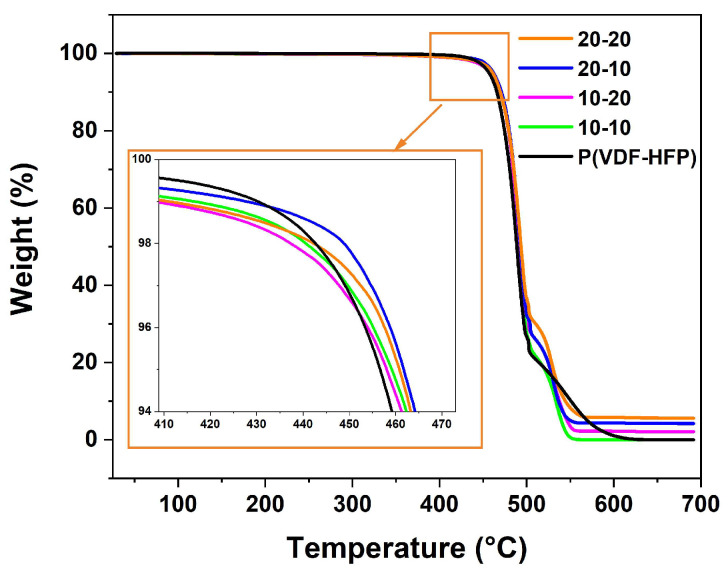
TGA thermograms in air of pure P(VDF-*co*-HFP) and 10-10, 10-20, 20-10, and 20-20 nanocomposites.

The thermal stability of both the pristine P(VDF-*co*-HFP) copolymer and the nanocomposite films was investigated under ambient conditions using thermogravimetric analysis (TGA). The thermograms obtained are presented in Figure 4, while Table 2 provides a summary of the initial degradation temperature (*T*_d_) and weight losses at 650 °C.

The pristine P(VDF-*co*-HFP) copolymer exhibited degradation starting from 446 °C, with a weight loss of 99.9% at 650 °C. Conversely, the PVDF-*g*-BT/P(VDF-*co*-HFP) films, prepared from 10-10, 10-20, 20-10, and 20-20 nanocomposites, demonstrated higher starting degradation temperatures, measuring 459 °C, 460 °C, 462 °C, and 464 °C, respectively, under similar conditions. In our case, with a moderate quantity of PVDF-*g*-BT-modified nanoparticles, the thermal stability of the double-shell nanocomposites was improved. Comparatively, an increase in *T*_d_ from 13 to 18 °C was observed compared to the pristine P(VDF-*co*-HFP). This improvement in thermal stability can be attributed to the presence of well-dispersed PVDF-*g*-BT core nanocomposites in the P(VDF-*co*-HFP) matrix. Dispersed nanoparticles act as barriers, hindering the permeability of volatile degradation compounds, thus delaying the liberation of thermal degradation components compared with pristine polymers [46]. Furthermore, in nanocomposites with the same fraction of P(VDF-*co*-HFP) (e.g., 10-10 and 20-10 samples), an increase in decomposition temperature was observed for PVDF-*g*-BT fillers with a higher BaTiO_3_ content (20%) compared to those with a lower fraction (10%). This increase can be attributed to improved dispersion and higher loading of the nanofiller. Previous studies conducted by our team [35] reported that the starting degradation temperatures of PVDF-*g*-BT 10% and PVDF-*g*-BT 20% were approximately 407 °C and 415 °C, respectively.

### 3.4. Differential Scanning Calorimetry (DSC)

**Figure 5:**
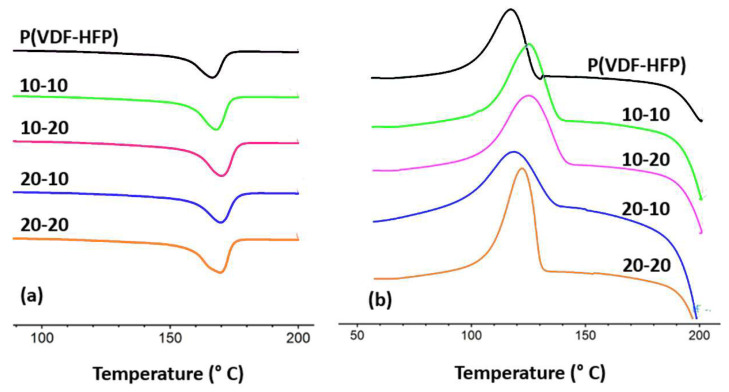
DSC thermograms of pristine P(VDF-*co*-HFP) and 10-10, 10-20, 20-10, 20-20 nanocomposite films: (**a**) second heating process and (**b**) second cooling process. Negative peaks are endotherms. Thermograms are shifted vertically for clarity.

DSC was employed to determine how the concentration of P(VDF-*g*-BT) nanocomposites affected the level of crystallinity and melting point of the nanocomposite films. The second heating and cooling DSC thermograms of our samples, obtained after a complete melting/recrystallization cycle, are presented in Figure 5a,b, respectively. The melting enthalpy (Δ*H*_m_), crystallization temperature (*T*_c_), melting temperature (*T*_m_), and crystallinity (*χ*) of each sample are summarized in Table 3.

Figure 5a shows a single endothermic melting peak for each film. As anticipated, the melting temperature (*T*_m_) of the pristine P(VDF-*co*-HFP) copolymer is 167 °C, as the low HFP content of 3 mol% slightly reduces the melting temperature of the copolymer below the 170 °C observed for PVDF alone [47]. The melting enthalpy (Δ*H*_m_) increases from 19 J/g for the pristine P(VDF-*co*-HFP) copolymer to 22 J/g and 26 J/g when introducing 10 wt% and 20 wt% of P(VDF-*g*-BT) loaded with 20 wt% of BaTiO_3_, respectively.

Also, the crystallization temperature (*T*_c_) of P(VDF-*co*-HFP) is 117 °C (Figure 5b), which rises to 125 °C for the 10-10 nanocomposite. Furthermore, the crystallinity of the films increases from 18% for the pristine copolymer to 25% after incorporating 20 wt% of PVDF-*g*-BT loaded with 20 wt% of BaTiO_3_. This suggests that the P(VDF-*g*-BT) core–shell nanofiller provides numerous heterogeneous nucleation sites within the P(VDF-*co*-HFP) matrix, thereby enhancing crystallinity. The increase in melting and crystallization temperatures upon introducing fillers into the P(VDF-*co*-HFP) matrix has been observed in previous studies [29,48,49]. For example, Zhang et al. [48] prepared core–shell-structured BaTiO_3_@polyalinine nanoparticles embedded in a P(VDF-*co*-HFP) matrix and noted an increase in T_c_ for small filler amounts, along with a higher value of crystallinity. They explained this behavior as the interplay of two factors: (i) the introduction of nanofillers into the P(VDF-*co*-HFP) matrix provides additional nucleation sites, promoting crystallinity, and (ii) the fillers can impede the movement of P(VDF-*co*-HFP) chains, delaying the crystallization process and resulting in an observed increase in crystallization temperature.

### 3.5. Dielectric Properties

The molecular dynamics of both the P(VDF-*co*-HFP) copolymer matrix and its core–double-shell nanocomposites were examined using broadband dielectric spectroscopy (BDS), which enables the characterization of their behavior with respect to temperature and frequency. This technique measures the electrical impedance and provides information about the dielectric permittivity, dielectric loss, and electrical modulus [50]. These parameters are crucial for assessing the enhancement of permittivity, conductivity, and other electrical characteristics relevant to the intended applications of the materials.

#### 3.5.1. Dielectric Properties of P(VDF-*co*-HFP) Matrix

**Figure 6:**
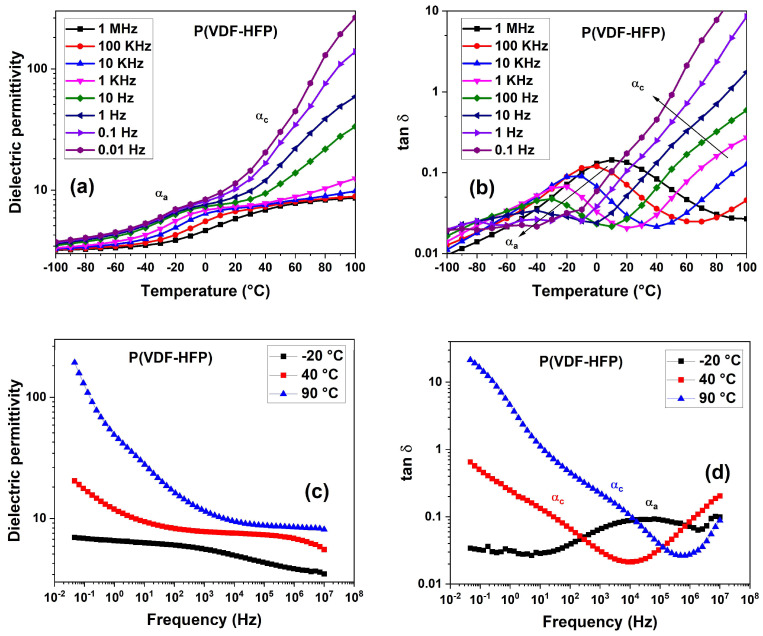
Dielectric permittivity (*ε*′) and loss tangent (tan *δ*) of P(VDF-*co*-HFP) versus temperature at different frequencies (**a**,**b**) and versus frequency at selected temperatures (**c**,**d**). The label *α*_a_ indicates both *β* and *α*_a_ relaxation (see text).

Figure 6, which depicts changes in the dielectric permittivity (*ε*′) and dissipation factor (tan *δ* = *ε*″/*ε*′) as a function of temperature and frequency, exemplifies the dielectric relaxation behavior of P(VDF-*co*-HFP).

Two distinct relaxation maxima are identified for P(VDF-*co*-HFP) that are close to and higher than the glass transition temperature (*T*_g_, around −36 °C as measured) (Figure 6a,b). Secondary relaxation, denoted as *β* relaxation, is the only one observable at low temperatures. Moreover, the dielectric relaxation arising from segmental motions within the amorphous phase is observed in the temperature range of −40 °C to around 0 °C and is referred to as primary or glass–rubber relaxation (α_a_) [51]. This relaxation originates from the micro-Brownian motion of polymer chains, causing long-range dipole relaxation motions. When the temperature reaches the glass transition temperature, the dipoles become sufficiently mobile to realign themselves with the applied electric field, leading to an increase in permittivity. *β* and α_a_ relaxation partially overlap, as reported in previous studies on PVDF [40]. Hence, the combined relaxations observable at lower temperatures are denoted here as *β*/α_a_.

The real part of the dielectric function, or permittivity, *ε*′, has a point of inflection at the lowest frequencies, connected to an additional relaxation process, known as α_c_ relaxation. This process is related to relaxation occurring within the crystalline phase of P(VDF-*co*-HFP), and shows up at temperatures around 0 to 40 °C [51,52].

Furthermore, a distinct contribution from conductivity, *σ*_dc_, can be observed on the low-frequency side of the tan *δ* plot (Figure 6d). This conductivity is due to the drift of ionic impurities under the action of the applied electric field, leading to an upturn in the tan *δ* value at high temperatures, as visible, for instance, from the value of tan *δ* (10.5) at 0.1 Hz and 90 °C in Figure 6d. This conduction mechanism can give rise to a phenomenon of electrode polarization (EP) [53], as well as to interfacial polarization known as Maxwell–Wagner–Sillars (MWS) polarization, that occurs at nanoscale phase segregations such as the ones between amorphous and crystalline regions of the polymer [54], as well as between the polymer and nanoscale inclusions in nanocomposites. Conductivity effects are evidenced by a rapid increase in *ε*′ at low frequencies, as visible, for instance, in Figure 6c, where *ε*′ = 100 at 0.1 Hz and 90 °C. As mentioned above, this mechanism is associated with the presence of free charge carriers, typically ions, that can drift under the applied electric field toward the electrodes, and/or accumulate around the various interfaces present in the sample. Macroscopic polarization, which appears much larger than the polarization occurring in the bulk of the material, can result from such charge accumulation. We note that such a conductivity contribution can overlap with that of α_c_ relaxation, making it difficult to discriminate the different relaxations. 

Figure 6a shows that in the glassy state, that is, below the glass transition temperature, the dipolar orientation is mostly impeded. The dielectric constant values of the P(VDF-*co*-HFP) copolymer at such temperatures are low, and rather independent of both temperature and frequency. In this case, high-frequency permittivity, *ε*′_∞_, dominates the dielectric constant. For instance, *ε*′_∞_ ≈ 3.5 at 10 Hz and −100 °C, which is a common value for atomic and electronic polarization in polar materials. The dielectric permittivity of P(VDF-*co*-HFP) at temperatures higher than T_g_, that is, in the temperature range of −20 °C to 40 °C, increases with increasing temperature and decreasing frequency. At room temperature, it amounts to around 8 at 1 kHz (Figure 6a), which is consistent with the values known from the literature (*ε*′~10), depending on the HFP content in the P(VDF-*co*-HFP) copolymer [52,55,56]. This permittivity increase is attributed to the enhanced segmental motions of dipole units within P(VDF-*co*-HFP), associated with both α_a_ and α_c_ relaxation.

#### 3.5.2. Dielectric Properties of Core–Double-Shell PVDF-g-BT/P(VDF-*co*-HFP) Nanocomposites

**Figure 7:**
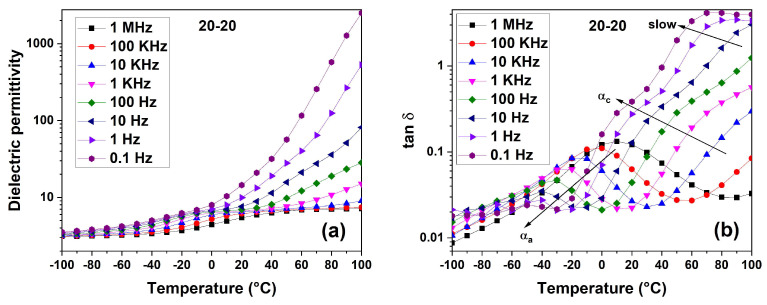
Dielectric permittivity, ε′ (**a**) and loss tangent, tan δ (**b**) of the 20-20 nanocomposite film versus temperature at different frequencies. The label *α*_a_ indicates both *β* and *α*_a_ relaxation (see text).

The dielectric properties of the nanocomposites were also measured as a function of frequency and temperature, exhibiting similar trends across all samples. Figure 7 illustrates the variation in *ε*′ and tan *δ* of the 20-20 nanocomposite film with temperature at different frequencies (plots for samples 10-10, 10-20, and 20-10 can be found in Figure S4 of the Supporting Information). At low frequencies, the real part of permittivity (*ε*′) displays two inflection points associated with two relaxation processes: *β*/α_a_ around the glass transition temperature (T_g_) (−40 °C to 0 °C) and α_c_ above T_g_ (0 °C to 60 °C). These relaxations correspond to the dipole relaxation phenomena occurring in the amorphous and crystalline phases of the polymer matrix, respectively. Figure 7b presents the loss tangent (tan *δ*) as a function of temperature and frequency, with arrows indicating the evolution of these processes. The relaxation peak related to the *β*/α_a_ process shifts to higher temperatures with increasing frequency. Notably, tan *δ* exhibits an inflection point corresponding to α_c_ relaxation, which is partly masked by the conductivity contribution arising from electrode polarization and/or the interfacial Maxwell–Wagner–Sillars (MWS) relaxation occurring at high temperatures and lower frequencies. This “slow” process is responsible for the significantly higher values of *ε*′ observed at high temperatures and low frequencies (e.g., *ε*′ = 1000 at 0.1 Hz and 90 °C, Figure 7a). The dielectric permittivity increases with temperature for all samples. For instance, at 1 Hz and 60 °C, the dielectric permittivity increases to 20 for P(VDF-*co*-HFP) (Figure 6), while in the 20-20 nanocomposite, it reaches a value close to 40 (Figure 7a). This rapid increase in the dielectric constant with temperature is likely attributed to the enhanced interfacial polarization effect [54].

**Figure 8:**
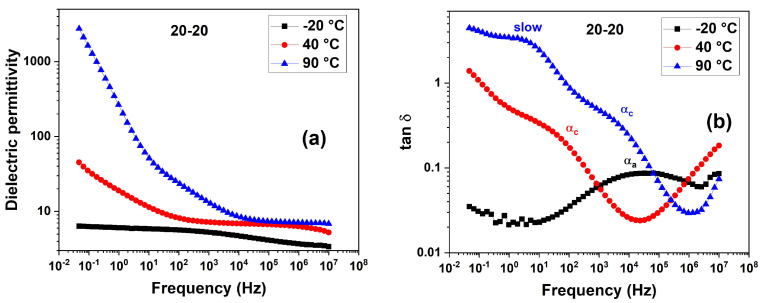
Dielectric permittivity, *ε*′ (**a**) and loss tangent, tan *δ* (**b**) of the 20-20 nanocomposite film versus frequency at −20 °C, 40 °C, and 90 °C. The label *α*_a_ indicates both *β* and *α*_a_ relaxation (see text).

Figure 8 presents selected representative isothermal spectra of *ε*′ and tan *δ* for the 20-20 nanocomposite film. The corresponding spectra for the 10-10, 10-20, and 20-10 nanocomposite films can be found in Figure S5 of the Supporting Information. The selected temperatures for these spectra are −20 °C, 40 °C, and 90 °C, which are near or above the glass transition temperature (T_g_).

Electrode and interfacial polarization resulting from conductivity dominate in the lower frequency range, leading to significantly high values of *ε*′. This phenomenon is particularly noticeable at higher temperatures, as observed in the 90 °C isotherm. Similar isotherms, however, exhibit a plateau accompanied by a decrease in *ε*′ at greater frequencies (Figure 8a). Due to the polar segments’ rotational motion within the material, which prevents them from following the applied electric field, the dielectric permittivity decreases with increasing frequency.

At 90 °C, the plateau in *ε*′ is broad and centered around 200 kHz, which falls within the frequency range where conductivity effects are not prominent. In contrast, at lower temperatures, the same plateau shifts to lower frequencies. Additionally, a second step in *ε*′ with frequency appears, signifying the existence of *α*_c_ relaxation. The findings reported for the reference polymer P(VDF-*co*-HFP) are consistent with this behavior. At −20 °C, large frequencies of *β*/α_a_ relaxation are seen, but low frequencies of α_c_ relaxation are observed within our spectral window. Figure 8b’s representation of the plot of tan *δ* as a function of frequency might be used to gain a more thorough understanding, although with varied temperature dependencies, the contributions of *β*/α_a_ and *α*_c_ relaxation exhibit a trend towards lower frequencies as the temperature drops. As the glass transition temperature is approached, these relaxations are likely to combine.

The significant increase in dielectric permittivity observed at 90 °C can be attributed to the presence of a slow process associated with enhanced interfacial polarization effects resulting from conduction. This effect primarily manifests in the low-frequency range, as interfacial polarization takes longer to develop compared to other types of processes. The existence of this slow process at low frequency and high temperature (T = 90 °C) is also shown in Figure 8b. The significant differential in electrical conductivity between the nanofillers and the copolymer matrix can be used to explain this phenomenon. It causes an increase in charge carriers at the interfaces where the different components of the nanocomposite film come together, which causes noticeable polarization and, as a result, a high dielectric constant [57].

**Figure 9:**
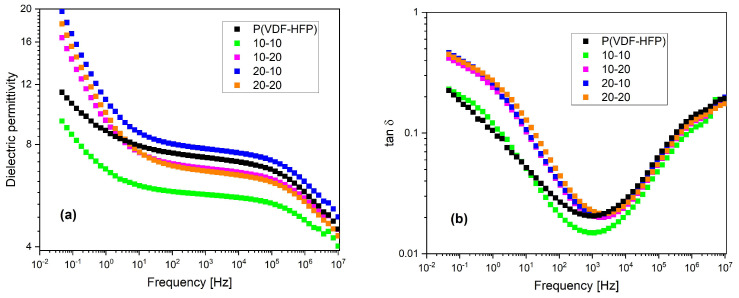
Frequency dependence of (**a**) dielectric permittivity and (**b**) dielectric loss tangent for the pristine P(VDF-*co*-HFP) copolymer and the core-double shell structured nanocomposites, recorded at 20 °C.

Figure 9 compares the four core–double-shell-structured nanocomposites to the pristine P(VDF-co-HFP) copolymer, showing the frequency dependence of dielectric permittivity and dielectric loss tangent recorded at 20 °C. At this temperature, the value of the plateau in the frequency range of 10^2^ to 10^5^ Hz is due to the contribution of both the BT nanoparticle inclusion and the polymer (*β* and α_a_ process), while the contribution of crystalline relaxation α_c_, as well as of the conductivity effects, starts at higher temperatures. We notice that the dielectric constant and the BT fraction do not show the same trend, because of the contribution of the amorphous polymer fraction, which should be determined quantitatively to rationalize the obtained results. However, a qualitative analysis can be conducted by assuming that the crystallinity of P(VDF-*co*-HFP) has similar values among the different core–double-shell nanocomposites, which should be acceptable as a first assumption, since it was observed via DSC that the crystallinity of fully melted and recrystallized nanocomposite samples ranged between 22% and 25%. In Table 4, the volume fractions ϕ in the core–double-shell nanocomposites of BT, of PVDF in PVDF-g-BT, and of P(VDF-*co*-HFP) are reported. Such fractions have been determined by solving the following linear equation system:

(2) $$x=\frac{\phi_{BT}}{\phi_{BT}+\phi_{PVDF}}\;\frac{1-X}{X}=\frac{\rho_{P(VDF-co-HFP)}\phi_{P(VDF-co-HFP)}}{\rho_{PVDF}\phi_{PVDF}+\rho_{BT}\phi_{BT}}\;\phi_{BT}+\phi_{PVDF}+\phi_{P(VDF-co-HFP)}=1$$

where ρ_BT_ = 6.02 g/cm^3^, and ρ_P(VDF-*co*-HFP)_ ~ ρ_PVDF_ = 1.78 g/cm^3^.

The highest value of dielectric constant is obtained for the 20-10 nanocomposite, which has an intermediate BT fraction (1.1 vol %, consistent with the BT fraction herein derived via TGA, that is, 1.3 vol %, within error; see Table 2), along with the highest fraction of P(VDF-*co*-HFP) (92.5 vol %). Since the amorphous fraction of P(VDF-*co*-HFP) is expected to be much higher than that of the PVDF fraction belonging to the PVDF-*g*-BT nanocomposite, which instead tends to be mostly crystalline [40], this result looks qualitatively consistent with expectations. For the same reasons, the lowest dielectric constant is obtained for the 10-10 sample, which has the smallest BT content (0.5 vol %), although the P(VDF-*co*-HFP) fraction (91.0 vol %) is almost as high as for the 20-10 sample. Finally, for the other two samples (10-20 and 20-20), there is a balance between the effects of the two components (BT and amorphous polymer), which provides intermediate results for the dielectric constant. The rather high value of the dielectric constant of pristine P(VDF-*co*-HFP) compared to the core–double-shell nanocomposites could be ascribed to its smaller crystallinity value, due to the absence of crystallization sites provided by the addition of PVDF-*g*-BT to the nanocomposites.

Similar non-monotonic trends of the dielectric constant with nanoparticle fractions have been observed in other studies [58,59,60]. For example, Khodaparast et al. [60] assembled nanocomposites by incorporating three different nanoparticles, namely, titania, silica, and alumina, into a PVDF matrix using a solution blending and casting method. These authors demonstrated that even when the fillers exhibited higher dielectric permittivity than PVDF alone, the resulting nanocomposites could exhibit a lower dielectric constant compared to the pure polymer. They highlighted the significance of the chemistry and interactions between the fillers and PVDF, which can play a more crucial role than the dielectric permittivity of the filler itself in determining the dielectric permittivity of the resulting nanocomposite.

Electrode and/or interface polarization can be the cause of the quick rise in permittivity at low frequencies. Other low-frequency relaxations may be completely eclipsed by this phenomenon (Figure 6, Figure 7 and Figure 8) [61]. To address this issue, the concept of an electric modulus was introduced, which helps mitigate the influence of conductivity-induced polarization [61,62,63,64]. The definition of the electric modulus, abbreviated as M, is as follows:

(3) $$M^{*}=\frac{1}{\varepsilon^{*}}=M^{\prime }+iM^{\prime \prime }=\frac{\varepsilon^{\prime }}{\varepsilon^{\prime 2}+\varepsilon^{\prime \prime 2}}+i\;\frac{\varepsilon^{\prime \prime }}{\varepsilon^{\prime 2}+\varepsilon^{\prime \prime 2}}$$

where the real and imaginary parts of the electric modulus, respectively, are denoted by *M*′ and *M*″, and the real and imaginary parts of the dielectric function are denoted by *ε*′ and *ε*″, respectively. To examine the bulk relaxation properties, the imaginary part of the electric modulus, sometimes referred to as Modulus″ or *M*″, is frequently used in the form of loss curves [57]. This approach is favored because conductivity effects are observed as a dielectric peak rather than an increase at low frequencies. 

**Figure 10:**
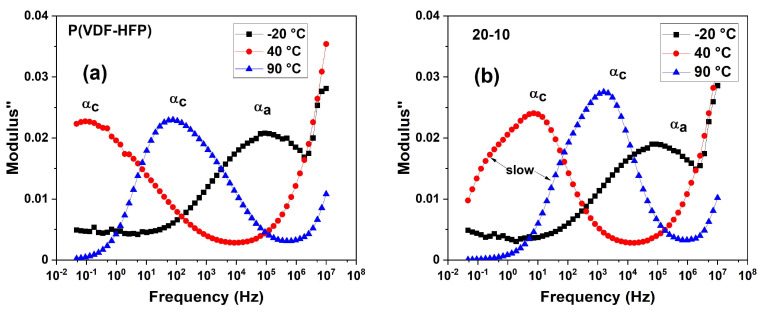
Modulus M″ versus frequency at selected temperatures (−20 °C, 40 °C, and 90 °C) for the P(VDF-*co*-HFP) matrix (**a**) as well as the corresponding 20-10 nanocomposite (**b**). The label *α*_a_ indicates both *β* and *α*_a_ relaxation (see text).

Figure 10 illustrates the variation in *M*″ with frequency at different temperatures (−20 °C, 40 °C, and 90 °C) near and above T_g_ for both the P(VDF-*co*-HFP) matrix and the corresponding 20-10 nanocomposite. In Figure S6 of the Supporting Information, the dielectric loss modulus (*M*″) of P(VDF-*co*-HFP) and the nanocomposite films (10-10) is presented as a function of frequency in the temperature range of 0 °C to 100 °C.

A notable difference is observed between the P(VDF-*co*-HFP) copolymer and the nanocomposite films. In contrast to the copolymer, which shows two relaxation peaks, nanocomposite films show a third relaxation process, which is sometimes referred to as a “slow” process in line with earlier research [40,65]. The accumulation of free charges at the interfaces between the fillers and the polymer matrix [54,66,67] is what causes this “slow” process, which is frequently linked to interfacial polarization [65].

The *M*″-versus-frequency behavior of the 20-20 nanocomposite film at various temperatures is presented in Figure S7 of the Supporting Information. These findings are consistent with the matrix’s and associated nanocomposites’ dynamic molecular mobility, which was previously studied in terms of permittivity and tan *δ*.

**Figure 11:**
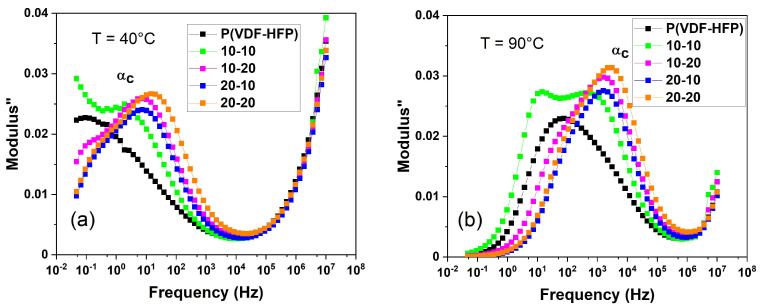
Modulus M″ versus frequency for the P(VDF-co-HFP) matrix and the corresponding nanocomposite films at the selected temperatures of 40 °C (**a**) and 90 °C (**b**).

Figure 11 provides a comparison of *M*″ between P(VDF-*co*-HFP) and its nanocomposites at 40 °C and 90 °C, showcasing both the relaxation in the crystalline phase and the slow relaxation observed in the nanocomposites.

To perform a quantitative analysis of the dielectric relaxations, the Havriliak–Negami (HN) function is employed:

(4) $$\varepsilon^{*}\left(f\right)={\varepsilon_{\infty }+\Sigma }_{k}\left\lfloor \frac{∆\varepsilon_{k}}{{\left(1+{\left(i\frac{f}{f_{0k}}\right)}^{a_{k}}\right)}^{b_{k}}}\right\rfloor +\frac{\sigma_{dc}}{{\varepsilon_{0}(i2\pi f)}^{n}}$$

where the index *k* denotes the considered relaxation process (α_c_ and α_a_), *f*_0*k*_ is the related relaxation frequency, ∆*ε_k_* is the dielectric relaxation strength, defined as ∆*ε* = *ε*_U_ − *ε*_R_ (ε_U_ and *ε*_R_ are the low (unrelaxed)- and the high (relaxed)- frequency limits of the real part of dielectric permittivity with respect to the relaxation frequency range, in our case, *ε*_R_ = *ε*_∞_), and *a_k_* and *b_k_* are the parameters describing the distribution width and asymmetry of relaxations, respectively [52]. The conductivity contribution is also considered in this fitting function, where *σ*_dc_ is the dc conductivity and *n* is a conductivity exponent to model different conduction mechanisms. All fittings were performed on the *ε*″ (dielectric loss) data to the imaginary part of Equation (4). The HN function is well suited to describing the dielectric relaxations of polymeric materials. The fitting procedure aims to determine appropriate values for the parameters *f*_0k_, ∆*ε*_k_, *a*_k_, and *b*_k_ by fitting the HN function to experimental data. The resulting values provide insights into the nature of the dielectric relaxation process and the underlying dynamics in the material being studied.

Table 5 summarizes the fitting parameters for the α_c_ relaxation process at T = 40 °C. This temperature was chosen because it is close enough to room temperature, and because an α_c_ relaxation peak is exhibited at such temperature within the explored frequency window, in order to allow reasonable fitting for the estimation of the relaxation parameters. We notice that the *α*_c_ relaxation-related increase in dielectric strength (Δ*ε*) is higher in the nanocomposites than in the pristine P(VDF-*co*-HFP) matrix. The lowest increment of Δ*ε* is found for the 20-10 nanocomposite, that has the lowest PVDF crystalline fraction, derived as the sum of the grafted PVDF fraction (considered as 100% crystalline) and the crystalline fraction of P(VDF-*co*-HFP) (considered as around 25% crystallinity). This finding is consistent with the assumption that *α*_c_ relaxation is related to the crystalline fraction. The width parameter *a* appears to be stable around 0.6, while the symmetry parameter *b* shows that the shape of relaxation in composites is more symmetric than in the pure polymer. Concerning *f*_0_ and ∆*ε*, the higher the crystalline volume fraction, the higher the dielectric relaxation strength associated with the *α*_c_ process.

The temperature dependence of thermally activated processes is described by the Arrhenius relation:

(5) $${\ln f_{0}}_{\;}=\ln f_{A}-\frac{E_{a}}{RT}$$

where *f*_A_ is the Arrhenius frequency for infinite temperature, *E*_a_ is the activation energy for the process, *R* is the gas constant, and *T* is the absolute temperature. This relationship is known to describe the relaxation frequencies of many dielectric processes.

**Figure 12:**
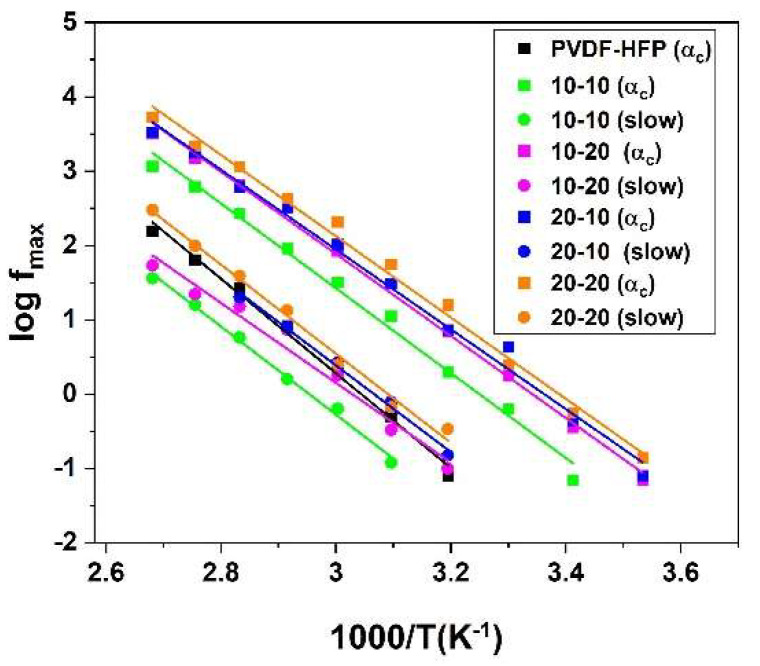
Variation in logarithmic peak frequency as a function of inverse temperature for pristine P(VDF-*co*-HFP) copolymer and related nanocomposite films for α_c_ and slow processes.

Figure 12 exhibits the logarithmic relaxation frequency (Log *f*_0_) as a function of the inverse temperature (1000/T) for the α_c_ and slow processes. This particular kind of Arrhenius plot is known as a relaxation plot. A relaxation process exhibiting a linear trend on a relaxation plot is referred to as having Arrhenius behavior. Both processes evidence an Arrhenius trend; therefore, fitting the data with the Arrhenius relationship of Equation (5) provides the Arrhenius frequency and the activation energy in each case. The results are summarized in Table 6.

We can conclude that the P(VDF-*co*-HFP) copolymer exhibits slower relaxation, while nanocomposites with lower activation energies demonstrate faster relaxation processes, by comparing the activation energy values of the various samples.

**Figure 13:**
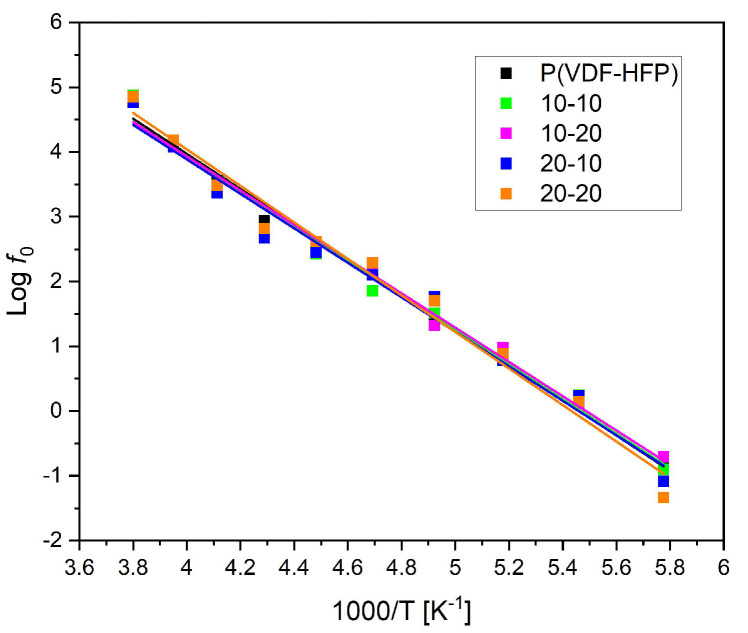
The logarithmic relaxation frequency variation as a function of inverse temperature for pristine P(VDF-*co*-HFP) copolymer and related nanocomposite films for the *β* process, with deviation from Arrhenius behavior due to the α_a_ process (see text).

We chose the data obtained at lower temperatures (−100 °C to −10 °C) and fitted the dielectric loss data using Equation (4) for all samples in order to quantify *β* and α_a_ relaxation. The outcomes are displayed in Figure 13. The obtained trend is approximately linear, denoting Arrhenius behavior, although with an inflection close to the expected glass transition temperature for all samples.

The Arrhenius parameters obtained by fitting the data from Figure 13 using Equation (5) are listed in Table 7. A lower Coefficient of Determination (COD) denotes higher deviation from the ideal Arrhenius behavior. The deviation occurs close to the expected glass transition temperature, likely caused by the influence of primary *α*_a_ relaxation. This relaxation seems essentially unaffected by the fraction of nanocomposites.

### 3.6. Mechanical Properties

**Figure 14:**
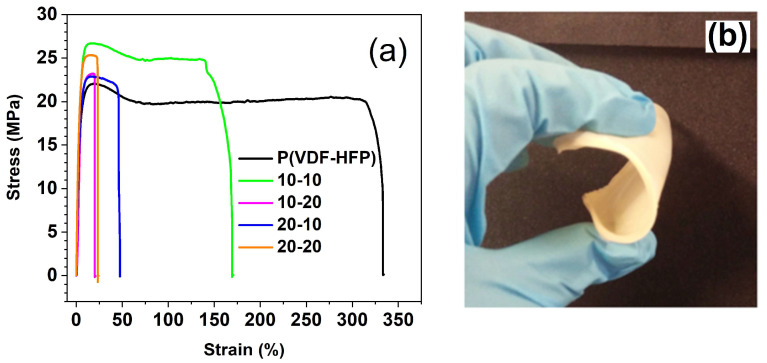
(**a**) Typical stress–strain curve of pristine P(VDF-*co*-HFP) copolymer and PVDF-*g*-BT@P(VDF-*co*-HFP) nanocomposite films and (**b**) photograph demonstrating the flexibility of the prepared nanocomposite films.

Through uniaxial tensile tests, the mechanical characteristics of the nanocomposite films were identified. The stress-versus-strain curves for both the pristine P(VDF-*co*-HFP) copolymer and the prepared nanocomposite films are depicted in Figure 14. The samples demonstrate ductile behavior, with the pristine copolymer exhibiting more pronounced ductility compared to the nanocomposite films, where the ductility is reduced.

**Figure 15:**
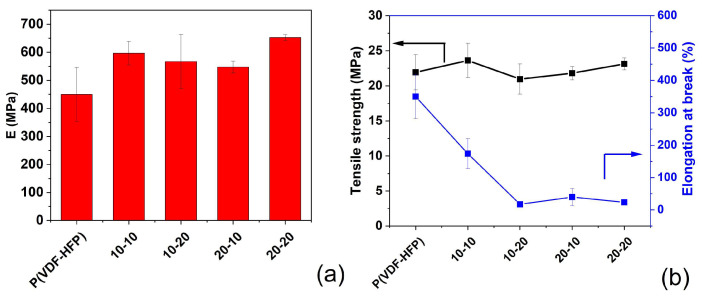
(**a**) Young’s modulus and (**b**) tensile strength with elongation at break for pristine P(VDF-*co*-HFP) copolymer and PVDF-*g*-BT@P(VDF-*co*-HFP) nanocomposite films.

During the testing procedure, four measurements were performed for each sample to accurately assess the key parameters defining the mechanical performance of the resulting films. The obtained values for Young’s modulus and tensile strength have been compiled and are depicted in Figure 15.

The P(VDF-*co*-HFP) copolymer displayed a tensile strength of 22 MPa, accompanied by an elongation at break value of approximately 500%. In the case of the 10 wt% P(VDF-*g*-BT) nanocomposite, incorporating 10 wt% of BaTiO_3_, the elongation at break decreased to approximately 100%, while the tensile strength increased to 24 MPa. As anticipated, a reduction in the elongation at break was observed in all the nanocomposite films compared to the pristine copolymer, in accordance with previous studies [68,69,70].

In terms of Young’s modulus, the materials can be ranked in decreasing order as follows: 20-20 > 10-10 > 10-20 > 20-10 > P(VDF-*co*-HFP). The addition of P(VDF-*g*-BT) nanocomposites to the P(VDF-*co*-HFP) matrix resulted in increased stiffness of the resulting films. This result is consistent with the results obtained by Ponnamma et al. [71], who prepared P(VDF-*co*-HFP) nanocomposites with BaTiO_3_ and hexagonal boron nitride using a casting method. Their mechanical measurements demonstrated an enhancement in both Young’s modulus and the tensile strength of the nanocomposites compared to the P(VDF-*co*-HFP) copolymer, along with a decrease in elongation at break. A similar trend was observed by Tarhini et al. [72], who incorporated graphene nanoflakes into a P(VDF-*co*-HFP) matrix. They observed an improvement in Young’s modulus for the nanocomposite films compared to the pristine copolymer film. This enhancement can be attributed to the restriction of segmental movements of P(VDF-*co*-HFP) chains due to the introduction of nanoparticles, as well as the uniform distribution of nanofillers, which form strong bonds with the matrix.

## 4. Conclusions

A series of double-shell PVDF-*g*-BT/P(VDF-*co*-HFP) nanocomposite films were synthesized via a solution mixing method using a pristine P(VDF-*co*-HFP) copolymer and P(VDF-*g*-BT) nanofillers. The obtained films were fully characterized using techniques such as FT-IR, SEM, TGA, and DSC. These analyses confirmed the successful incorporation and effective dispersion of P(VDF-*g*-BT) nanocomposites within the P(VDF-*co*-HFP) matrix, leading to improved thermal properties of the system. Broadband dielectric spectroscopy (BDS) investigations unveiled the presence of three dipolar and interfacial relaxation phenomena, which contributed to the observed increase in permittivity. Notably, the highest increase in the dielectric constant was achieved when utilizing the highest BT content in the filler combined with the lowest filler fraction. Mechanical analysis exhibited an improvement in Young’s modulus and the tensile strength of the nanocomposite films versus the pristine copolymer. Consequently, the films exhibited increased stiffness, further validating the beneficial impact of the PVDF-*g*-BT nanocomposites on the mechanical properties of the system.

The concentration of core–shell structures is proportional to the fraction of BT, while the volume of the PVDF shell is proportional to the fraction of PVDF. The trends of the various physical quantities investigated could be compared to either one or the other. For instance:

(i) The starting degradation temperature determined via TGA increases with the concentration of core–shell structures.
(ii) Within an acceptable error, Young’s modulus is higher for all nanocomposites than for the polymer alone and rises with the PVDF shell volume.
(iii) Tensile strength cannot be assigned a trend, because the error bars are comparable to the dispersion of data.
(iv) The elongation at break decreases with the core–shell structure concentrations.

Thanks to such interesting properties, these nanocomposite films have promising applications. One of the most suitable options, specifically, is to utilize them as electrolytes and binders for lithium-ion batteries [39,73].

## Figures and Tables

**Table 1:** Core–double-shell PVDF-g-BT x%/P(VDF-co-HFP) (X/Y) nanocomposite nomenclature.

Sample Composition PVDF-*g*-BT x%/P(VDF-*co*-HFP) (X/Y)	Code x-X	x% = BT/VDF Feed Mass inPVDF-*g*-BT *	X/Y Mass Ratio PVDF-*g*-BT x%/ P(VDF-*co*-HFP) in PVDF-*g*-BT/P(VDF-*co*-HFP)
10%/(10/90)	10-10	10	10/90
10%/(20/80)	10-20	10	20/80
20%/(10/90)	20-10	20	10/90
20%/(20/80)	20-20	20	20/80

* x% is the weight (feed) ratio applied during the RAFT polymerization of VDF to produce PVDF-*g*-BT [35].

**Table 2:** TGA weight losses and starting degradation temperatures *T*_d_ of the pure P(VDF-*co*-HFP) and its nanocomposite films.

Samples	*T*_d_ (°C)	Weight Loss (%) at 650 °C
P(VDF-co-HFP)	446	99.9
10-10	459	99.9
10-20	460	97.8
20-10	462	95.7
20-20	464	94.4

**Table 3:** Melting enthalpy (Δ*H*_m_), crystallization temperature (*T*_c_), melting temperature (*T*_m_) and crystallinity (*χ*) of P(VDF-*co*-HFP) copolymer and its nanocomposite films.

Samples	Δ*H*_m_ (J/g)	*T*_c_ (°C)	*T*_m_ (°C)	*χ* (%)
P(VDF-*co*-HFP)	19	117	167	18
10-10	23	125	168	22
10-20	24	125	170	23
20-10	22	119	170	21
20-20	26	122	170	25

**Table 4:** Volume fractions of the various components of the core–double-shell nanocomposites.

Samples	BT vol %	PVDF vol %	P(VDF-*co*-HFP) vol %
P(VDF-*co*-HFP)	0	0	100
10-10	0.5	8.5	91.0
10-20	1.0	17.2	81.8
20-10	1.1	6.4	92.5
20-20	2.4	13.1	84.5

**Table 5:** HN parameters for α_c_ relaxation at T = 40 °C for pristine P(VDF-*co*-HFP) and its corresponding nanocomposite films.

Samples	Log *f*_0_	Δ*ε*	*a*	*b*
P(VDF-co-HFP)	0.21	3.22	0.60	0.58
10-10	0.12	14.9	0.64	0.82
10-20	0.45	13.3	0.56	1
20-10	−0.06	5.3	0.61	0.77
20-20	0.03	15.7	0.54	1

**Table 6:** Arrhenius parameters of α_c_ and slow relaxations. COD stands for Coefficient of Determination, characterizing the quality of fitting.

Samples	Log *f*_A_		*E*_a_ (kJ mol^−1^)		COD	
	α_c_	Slow	α_c_	Slow	α_c_	Slow
P(VDF-*co*-HFP)	19.37	-	121.8	-	0.99	-
10-10	18.54	17.50	109.3	113.4	0.99	0.99
10-20	18.44	16.26	105.7	102.8	0.99	0.97
20-10	18.04	17.86	102.7	111.5	0.99	0.99
20-20	17.57	18.54	104.7	114.8	0.99	0.99

**Table 7:** Arrhenius parameters for *β*/*α*_a_ relaxation, obtained via fitting using Equation (5). COD stands for Coefficient of Determination, which characterizes the quality of fitting.

Samples	Log *f*_A_	*E*_a_ (kJ mol^−1^)	COD
P(VDF-co-HFP)	14.8 ± 0.4	52.0 ± 1.3	0.994
10-10	14.6 ± 0.6	50.8 ± 2.3	0.982
10-20	14.5 ± 0.5	50.6 ± 2.3	0.985
20-10	14.5 ± 0.7	50.83 ± 2.7	0.978
20-20	15.3 ± 0.7	53.9 ± 2.7	0.981

## Data Availability

The data presented in this study are available in the present article and in the related Supplementary Materials.

## Supplementary Materials

The following supporting information can be downloaded at: https://www.mdpi.com/article/10.3390/polym15143126/s1, Figure S1: ^19^F NMR spectrum of commercially available P(VDF-*co*-HFP) recorded in CDCl3; Scheme S1: Sketch illustrating the synthesis process of PVDF-*g*-BaTiO_3_ nanocomposites via RAFT polymerization of VDF in the presence of xanthates (from the modification of BaTiO_3_ nanoparticles), where TBPPi stands for *tert*-butyl peroxypivalate; Figure S2: Expansion of the −64 to −120 ppm region of the ^19^F HRMAS spectrum recorded in d6-DMSO of PVDF-*g*-BaTiO_3_ nanocomposite filled with 10 wt % of BaTiO3; Figure S3: Expansion of the −64 to −120 ppm region of the ^19^F HRMAS spectrum recorded in d6-DMSO of PVDF-*g*-BaTiO_3_ nanocomposite filled with 20 wt % of BaTiO_3_; Figure S4: Dielectric permittivity, ε′, and loss tangent, tan δ, of 10-10, 10-20, and 20-10 nanocomposite films versus temperature at different frequencies; Figure S5: Selected representative isothermal spectra recorded for ε′ and tan δ of the 10-10, 10-20, and 20-10 nanocomposite films; Figure S6: Frequency dependence of the dielectric loss modulus (Modulus′, or M″) of pristine P(VDF-*co*-HFP) and of its 10-10 nanocomposite film at different temperatures; Figure S7: Modulus M″ versus frequency at different temperatures of the 20-20 nanocomposite film.
