# Supplementary material for: Synthesis and Characterization of Core–Double-Shell-Structured PVDF-grafted-BaTiO3/P(VDF-co-HFP) Nanocomposite Films
Source: Polymers (Basel). 2023 Jul 22;15(14):3126. doi: 10.3390/polym15143126 (PMC10383315; doi:10.3390/polym15143126)
Supplement: Supplementary file 1 [file polymers-15-03126-s001.zip › polymers-2457327-supplementary.pdf]

SUPPORTING INFORMATION

Synthesis and characterization of core-double shell structured PVDF-*grafted*-$BaTiO_3$/P(VDF-*co*-HFP) nanocomposite films

Fatima Ezzahra Bouharras [a,b,c], Salima Atlas [a,d], Simone Capaccioli [b,e,f], Massimiliano Labardi [e,f,*], Abdelghani Hajlane [a], Bruno Ameduri [c], Mustapha Raihane [a,*]

[a] IMED-Lab, Faculty of Sciences and Techniques. Cadi Ayyad University (UCA), Av. A. El Khattabi, B.P. 549, 40000, Marrakesh, Morocco

[b] Dipartimento di Fisica, Università di Pisa, Largo Pontecorvo 3, 56127, Pisa, Italy

[c] ICGM, Univ. Montpellier, CNRS, ENSCM, 34293, Montpellier Cedex 5, France

[d] Polydisciplinary Faculty - Sultan Moulay Sliman University. Mghila P.O. 592, 23000, Béni-Mellal, Morocco

[e] CNR-IPCF, Sede Secondaria di Pisa, c/o Dipartimento di Fisica, Università di Pisa, Largo Pontecorvo 3, 56127, Pisa, Italy

[f] CISUP, Centro per l'Integrazione della Strumentazione dell'Università di Pisa, Lungarno Pacinotti 43/44, 56126, Pisa, Italy

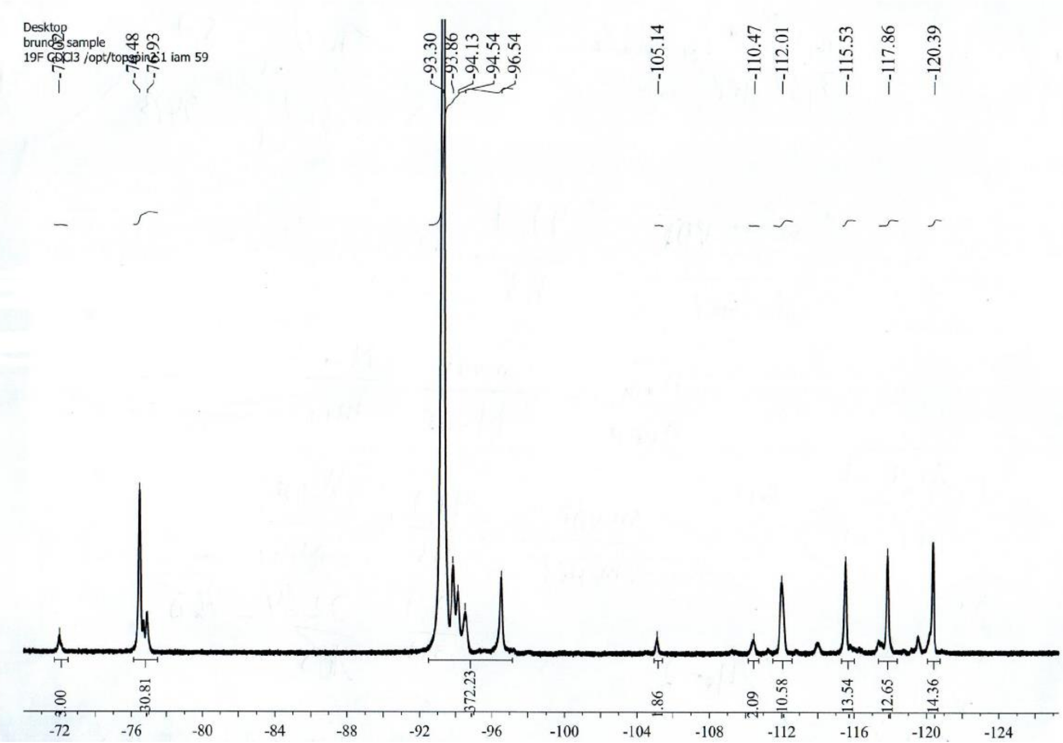

Figure S1. ^{19}F NMR spectrum of commercially available P(VDF-*co*-HFP) recorded in $CDCl_3$.

The VDF content was determined using Equation (1), and was estimated to be 97 mol %.

$$mol.\%\,VDF = \frac{\int_{-92.0}^{-119} VDF/2}{\int_{-92.0}^{-119} VDF/2 + \int_{-119.5}^{-120.5} HFP/2}$$

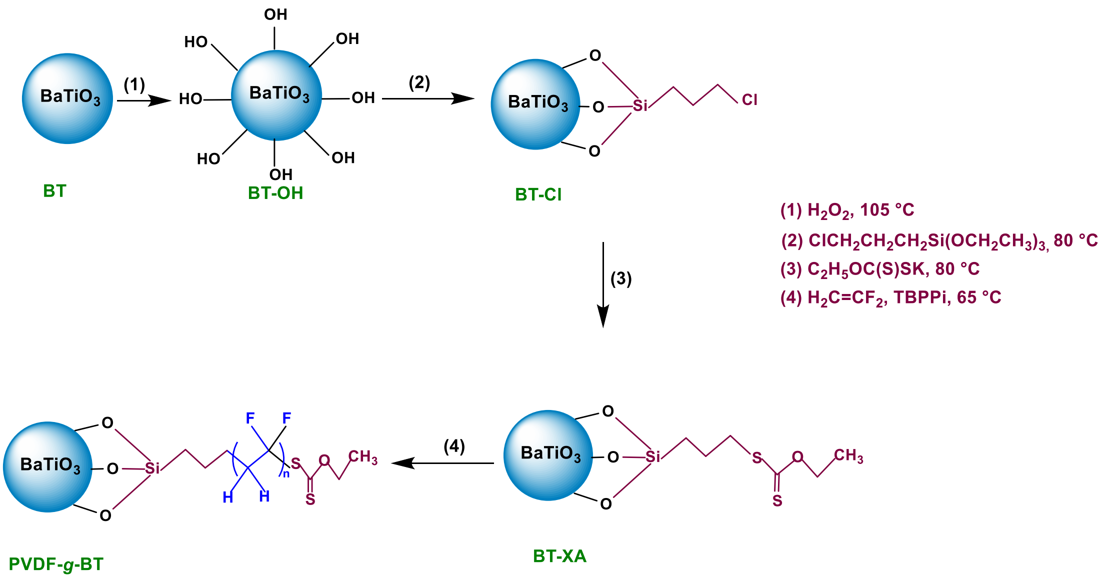

Scheme S1. Sketch illustrating the synthesis process of PVDF-*g*-BaTiO$_3$ nanocomposites by RAFT polymerization of VDF in the presence of Xanthates (from the modification of BaTiO$_3$ nanoparticles) where TBPPi stands for *tert*-butyl peroxypivalate [1].

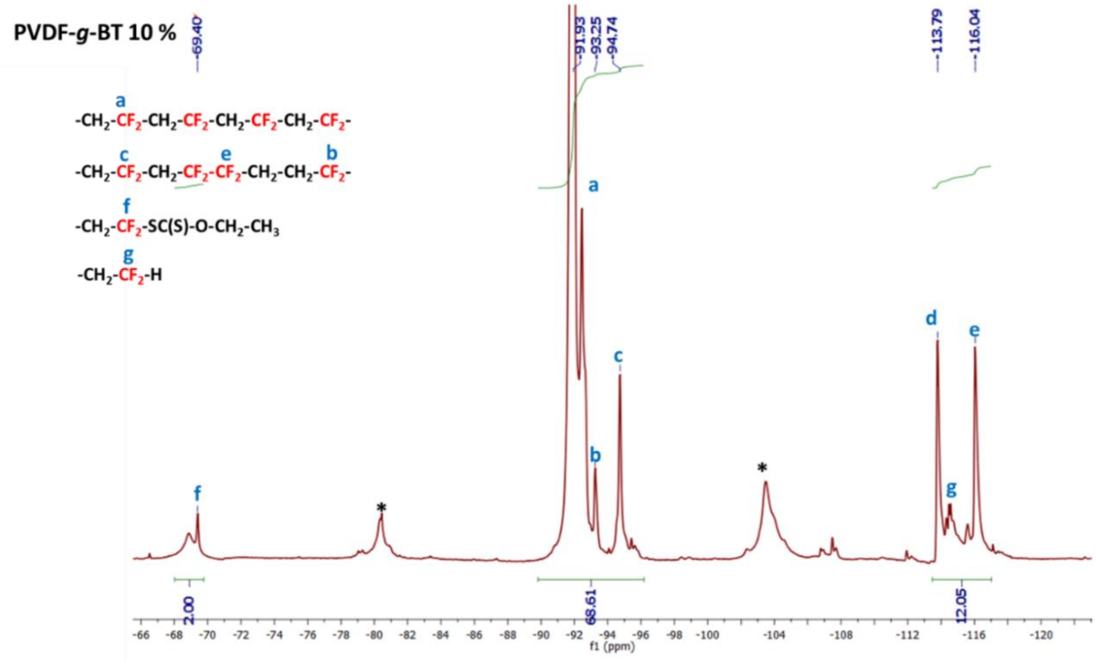

Figure S2. Expansion of the −64 to −120 ppm region of the ^{19}F HRMAS spectrum recorded in d$_6$-DMSO of PVDF-*g*-BaTiO$_3$ nanocomposite filled with 10 wt % of BaTiO$_3$ (* stands for the spinning bands).

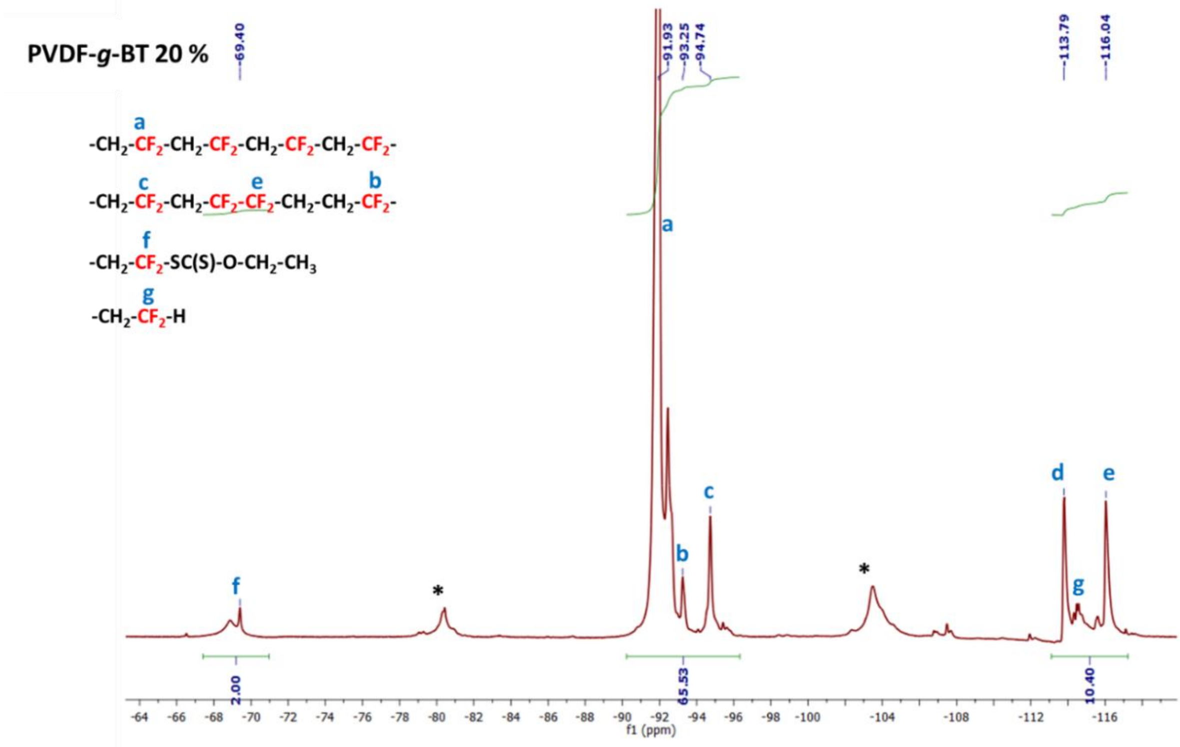

Figure S3. Expansion of the −64 to −120 ppm region of the ^{19}F HRMAS spectrum recorded in d_6-DMSO of PVDF-*g*-$BaTiO_3$ nanocomposite filled with 20 wt % of $BaTiO_3$ (* stands for the spinning bands).

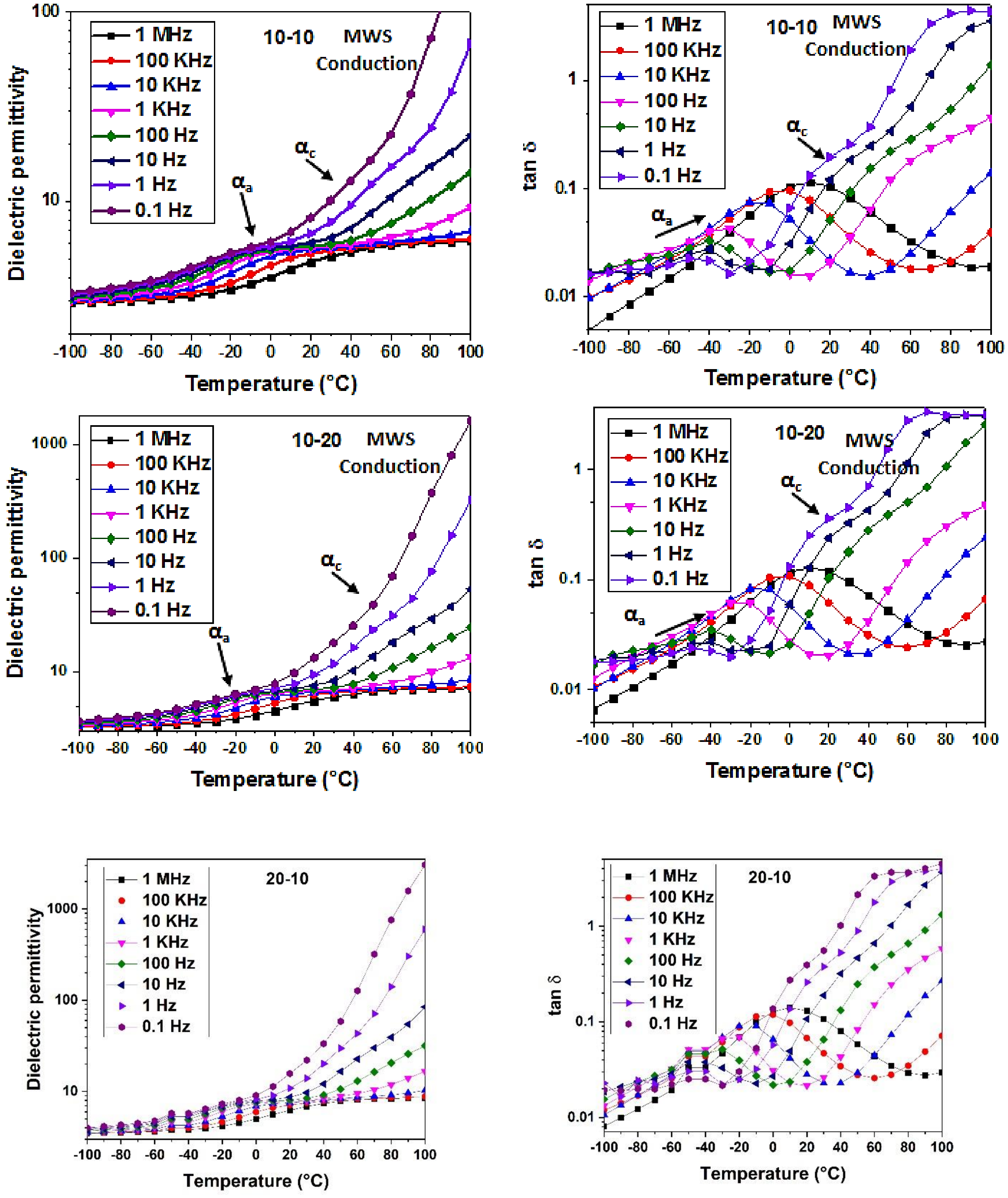

Figure S4. Dielectric permittivity, ε′, (left) and loss tangent, tan δ, (right) of 10-10, 10-20, and 20-10 nanocomposites films *versus* temperature at different frequencies.

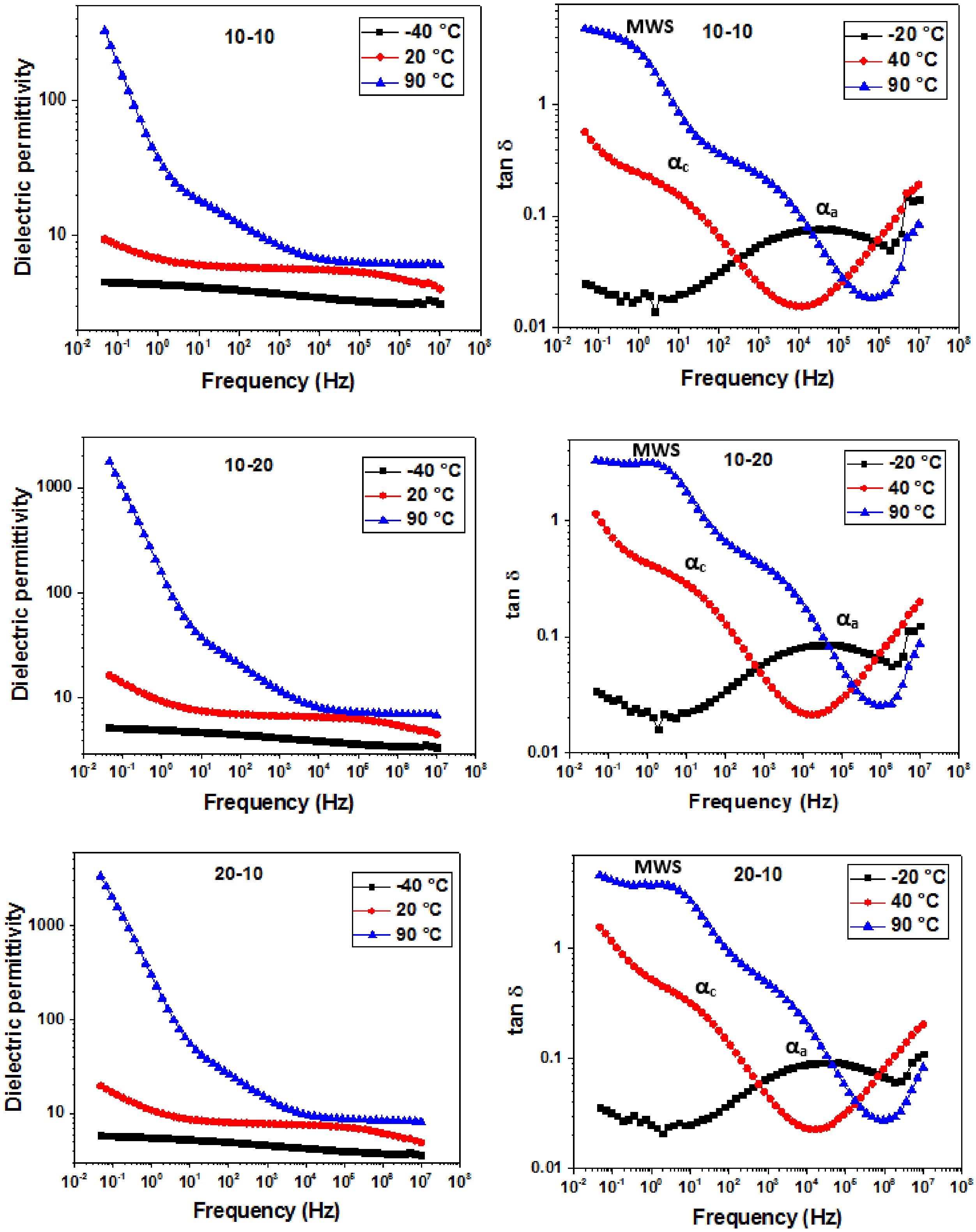

Figure S5. Selected representative isothermal spectra recorder for ε′ and tan δ of the 10-10, 10-20 and 20-10 nanocomposite films.

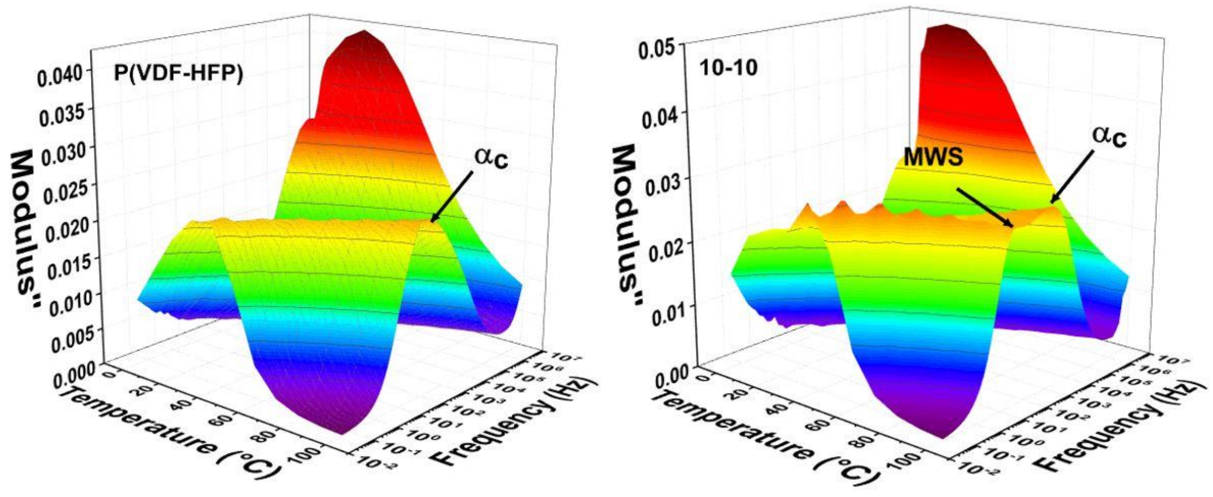

Figure S6. Frequency dependence of the dielectric loss modulus (Modulus", or M") of pristine P(VDF-*co*-HFP) and of its 10-10 nanocomposite film at different temperatures.

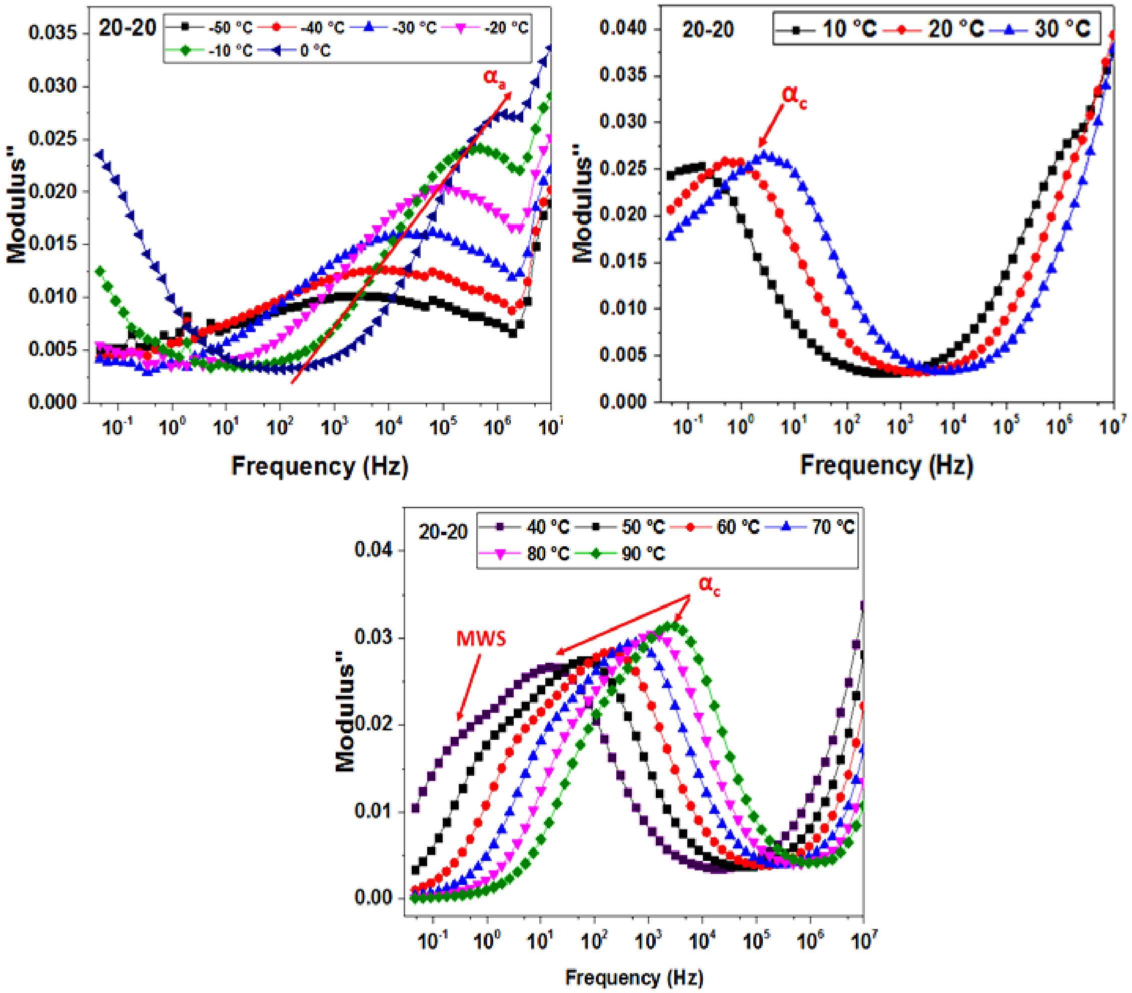

Figure S7. Modulus M" versus frequency at different temperatures of the 20-20 nanocomposite film.

Reference

[1] F.E. Bouharras, M. Raihane, G. Silly, C. Totee, B. Ameduri, Core shell structured Poly(Vinylidene Fluoride)-*grafted*-$BaTiO_3$ nanocomposites prepared via Reversible Addition fragmentation chain transfer (RAFT) polymerization of VDF for high energy storage capacitors, Polym. Chem. 10 (2019) 891–904. doi:10.1039/C8PY01706A.
